# When I say … haunted curriculum

**DOI:** 10.1111/medu.15537

**Published:** 2024-10-16

**Authors:** David A. Ansari, Neera R. Jain, Constance R. Tucker, Jennifer Karlin

**Affiliations:** ^1^ Department of Medical Education University of Illinois College of Medicine Chicago Illinois USA; ^2^ Centre for Medical and Health Sciences Education University of Auckland|Waipapa Taumata Rau Auckland New Zealand; ^3^ Education Improvement and Innovation Oregon Health & Science University Portland Oregon USA; ^4^ Family & Community Medicine University of California San Francisco California USA

## Abstract

As medical and health professions education (HPE) fields shift towards frameworks of justice, equity, diversity and inclusion (JEDI), there has been an increased focus on addressing the environments of learning to understand hierarchies of power and create better learning experiences for students who have historically been marginalised in these fields. We propose the *haunted curriculum* as an evocative conceptual framing to engage with the aspects of medical and HPE that are toxic and troubling to learners and to understand how violent histories continue to loom large and permeate the present. The *haunted curriculum* examines how forms of oppression and injustice, such as racism and ableism, are always ever‐present and often arise in unexpected ways during training. The *haunted curriculum* also helps to uncover how environments of training and professionalisation may themselves inflict these forms of oppression and injustice and may be ideal spaces to counter them.

## Abstract 2

The haunted curriculum encompasses aspects of health professional education that are toxic and troubling to learners while shining light on how violent histories loom large in the present.

## 1 INTRODUCTION

As medical and health professions education (HPE) shift towards frameworks of justice, equity, diversity and inclusion (JEDI), there has been an increased focus on addressing the environments of learning to understand hierarchies of power and create better learning experiences for students (e.g. Black, Indigenous and People of Colour [BIPOC], disabled and first‐generation people) who have been marginalised in these fields. Despite this focus, medical and HPE fields remain haunted for these learners. Learners encounter educational spaces that are inaccessible, experience material erasure when simulation manikins or textbooks depict light skin tones and exclude others, witness or experience distressing trauma, are evaluated according to antiquated notions of professionalism, and face bias from their peers, professors and patients. We propose the *haunted curriculum* as an evocative conceptual framing to engage with the aspects of medical and HPE that are toxic and troubling to learners and to understand how violent histories continue to loom large and permeate the present. Haunting provokes senses of incompleteness, disbelief, intractability and torment. Haunting also suggests that a lurking presence ought to be reckoned with—history rings in the present. The *haunted curriculum* examines how forms of oppression and injustice are always ever‐present and often arise in unexpected ways during training.

## 2 BEYOND THE HIDDEN CURRICULUM

The *haunted curriculum* bears a similar name to the *hidden curriculum*, a framework most notably associated with Hafferty and Franks that refers to the transmission of expertise through informal and implicit rules and values. The *haunted* is distinct from the hidden because it does not assume that some learning experiences are subtle or hidden. Rather, it demands we engage with both the tacit and obvious forms of oppression that linger in learning environments and harm some learners while leaving others unscathed. Learners of colour often perform additional labour of educating their peers and even faculty about forms of injustice and their health implications. Disabled learners find that notions of hyper‐ablebodiedness and mindedness are reinforced in HPE, which are exclusionary and undermine institutional commitments to JEDI. These learners may find that their peers or faculty do not take their concerns seriously. Like ghosts, not everyone sees these forms of oppression, and not everyone believes those who do. The haunted curriculum requires we understand that injustices are baked into the learning environment, such as when learners rotate through clinics for marginalised patient populations. These patients' vulnerabilities translate to learning experiences because learners witness first‐hand how structural determinants, such as poverty and immigration status, shape access to care.

The *haunted curriculum* responds to critiques of the hidden curriculum for emphasising revealing over taking responsibility for change.^1^ While the hidden curriculum paves the way for action, we contend that a deeper form of engagement is required in contexts of injustice in HPE, such as racism and ableism. The *haunted curriculum* requires us to sit with discomfort and engage with aspects of training that are pernicious, particularly for learners who have been unrecognised in HPE. Uncovering what is subtle does not effectively engage with the reasons why these fields have historically excluded marginalised learners and does not effectively engage with the reasons why marginalisation continues to dominate despite the growth of well‐intentioned JEDI‐oriented initiatives. We follow the wise words of Indigenous scholars Eve Tuck and C. Ree, who state that haunting is ‘the relentless remembering and reminding that will not be appeased by settler society's assurances of innocence and reconciliation’.^2^ ^, p. 642^ Might JEDI initiatives mask the reasons why these learners remain unrecognised? The *haunted curriculum* demands we conjure perspectives in HPE that have been erased, interrogate why those perspectives have been erased and understand how the forces that drive the erasure of these perspectives linger.

## 3 WHY HAUNTED?

Our introduction of haunting to HPE is inspired by the deeply abundant field of spectral studies and *hauntology*, a joining of haunting and ontology. While many associate hauntology with the late French philosopher Jacques Derrida, we also draw inspiration from Black Feminist and Indigenous scholars who offer depth to this notion of haunting, demonstrating non‐linear understandings of time and resistance to erasure. Viviane Saleh‐Hanna's Black Feminist Hauntology, which traces the relationship between colonial slavery and criminal justice violence to ‘capture the expanding and repetitive nature of structural violence’ and ‘locate a language to speak about actual, not just symbolic or theoretical violence’.^3^ ^, p. 8^ Similarly, within Indigenous studies, scholars detail how individuals and societies are haunted,^2^ with the notion of haunting offering a theoretical framework to subvert erasure.^4^ Haunting is more than a metaphor for witnessing and experiencing violence. Rather, it permits modes of remembering and apprehending forms of violence that accumulate over time. Haunting goes beyond an individual's experience and instead centres the collective as the denominator of experience. We must treat ghost stories as invitations to recognise student experiences as echoes of the past, calling us to remember past wrongs and identify why justice remains unfinished.

## 4 WHERE DO WE GO FROM HERE?

The *haunted curriculum* helps us understand how encounters in HPE settings, such as between learners and faculty or among learners, may produce and reproduce forms of oppression and injustice. Nevertheless, these encounters also offer rich opportunities to learn from these injustices and challenge these forms of oppression. Spectres of violent pasts materialise in all areas of the learning environment, reminding us of work that remains undone. Catherine Belling^5^ implores us to welcome rather than avoid ghosts, because they may not seek revenge as we so fear and instead be teachers who inspire us to engage the vestiges of our violent pasts. Learners who are members of communities that have been, and remain, subjects of such violence may see these ghosts most clearly, and thus we must pay attention to their sightings most pointedly. Responding to haunting involves taking responsibility to ensure physical spaces are accessible and not leaving it to disabled students to request accommodations, collectively calling out microaggressions and attending to the drivers of inequality that are often erased or are mischaracterized as cultural issues. When they tell us about these ghosts, we must believe them. It is time we tune in to long‐repressed hauntings, learn from them and move to action.

## AUTHOR CONTRIBUTIONS

**David A. Ansari:** Conceptualization; writing—original draft; writing—review and editing; project administration; formal analysis; supervision. **Neera R. Jain:** Conceptualization; writing—original draft; writing—review and editing; formal analysis. **Constance R. Tucker:** Conceptualization; writing—original draft; writing—review and editing; formal analysis. **Jennifer Karlin:** Conceptualization; writing—original draft; supervision; formal analysis; writing—review and editing.

## Data Availability

Data sharing not applicable to this article as no datasets were generated or analysed during the current study.
